# *Bartonella* spp. in Phlebotominae Sand Flies, Brazil

**DOI:** 10.3201/eid3010.240397

**Published:** 2024-10

**Authors:** Daniel Antônio Braga Lee, Paloma Helena Fernandes Shimabukuro, Andréia Fernandes Brilhante, Paulo Vitor Cadina Arantes, Gustavo Seron Sanches, Eliz Oliveira Franco, Rosangela Zacarias Machado, Ricardo G. Maggi, Edward B. Breitschwerdt, Marcos Rogério André

**Affiliations:** São Paulo State University, Jaboticabal, Brazil (D.A.B. Lee, P.V.C. Arantes, G.S. Sanches, E.O. Franco, R.Z. Machado, M.R. André);; Oswaldo Cruz Foundation, Belo Horizonte, Brazil (P.H.F. Shimabukuro);; Federal University of Acre, Rio Branco, Acre, Brazil (A.F. Brilhante);; North Carolina State University College of Veterinary Medicine, Raleigh, North Carolina, USA (R.G. Maggi, E.B. Breitschwerdt)

**Keywords:** Bartonella, phlebotomine, sand flies, Bartonellaceae, Phlebotominae, Carrion’s disease, Oroya fever, verruga peruana, vector, infections, vector-borne infections, Brazil

## Abstract

*Bartonella* spp. are opportunistic, vectorborne bacteria that can cause disease in both animals and humans. We investigated the molecular occurrence of *Bartonella* spp. in 634 phlebotomine sand fly specimens, belonging to 44 different sand fly species, sampled during 2017–2021 in north and northeastern Brazil. We detected *Bartonella* sp. DNA in 8.7% (55/634) of the specimens by using a quantitative real-time PCR targeting the 16S-23S internal transcribed spacer intergenic region. Phylogenetic analysis positioned the *Lutzomyia longipalpis* sand fly–associated *Bartonella gltA* gene sequence in the same subclade as *Bartonella ancashensis* sequences and revealed a *Bartonella* sp. sequence in a *Dampfomyia beltrani* sand fly from Mexico. We amplified a bat-associated *Bartonella nuoG* sequence from a specimen of *Nyssomyia antunesi* sand fly. Our findings document the presence of *Bartonella* DNA in sand flies from Brazil, suggesting possible involvement of these insects in the epidemiologic cycle of *Bartonella* species.

The genus *Bartonella* (Alphaproteobacteria: Bartonellaceae) comprises emergent and re-emergent opportunistic bacteria classified in 39 validated species (https://lpsn.dsmz.de/genus/bartonella), some of them capable of causing disease in both animals and humans (*1*). Mammals (e.g., rodents, bats, cats, dogs, ruminants), including humans, are the main reservoirs for bartonellae. The *Bartonella* species most often associated with disease in humans are *B. henselae* (the causative agent of cat scratch disease), *B. quintana* (the causative agent of trench fever), and *B. bacilliformis* and *B. ancashensis* (the causative agents of Carrion’s disease and verruga peruana) (*2*–*4*). Other species, including *B. clarridgeiae*, *B. koehlerae*, *B. vinsonii* subspecies *berkhoffii*, *B. elizabethae*, and Candidatus *Bartonella mayotimonensis*, also have been associated with disease in humans, especially in fever of unknown origin and culture-negative endocarditis cases (*5*,*6*). *Bartonella* spp. infect a variety of cells, including erythrocytes, pericytes, endothelial, dendritic, and macrophage cells and are associated with persistent intraerythrocytic bacteremia, suggesting a possible coevolution between these bacteria and their hosts, which may explain their remarkable adaptability to >1 mammal species (*2*,*7*,*8*). The ability of those bacterial species to maintain a persistent bacteremia over time dovetails with their main route of transmission, via bloodsucking arthropods (*9*). On the basis of molecular epidemiologic surveys and clinical observations, researchers have implicated many hematophagous arthropods in the transmission cycles of *Bartonella* spp.—mosquitoes (*9*), biting midges (*10*), triatomine bugs (*11*), mites (*12*,*13*), and flies (*14*)—adding to the list of those already identified as competent vectors (fleas, lice, phlebotomine sand flies, ticks) (*15*,*16*).

Phlebotomine sand flies (Diptera: Psychodidae: Phlebotominae) comprise >1,060 species, distributed worldwide, especially in tropical and subtropical regions (*17*). Given their hematophagous feeding habit, female sand flies are insects of considerable public health concern, because they act as vectors in the transmission of different pathogenic agents (bacteria, protozoa, virus), such as *Bartonella* sp., *Leishmania* sp., and Phleboviruses (*18*). Within the Bartonellaceae family, *B. bacilliformis* is notably the most important agent transmitted by phlebotomine sand flies. This *Bartonella* species is the causative agent of Carrion’s disease, which can manifest as 2 different syndromes (that can occur sequentially or independently): Oroya fever, characterized by an acute hemolytic anemia with an untreated fatality rate of up to 90%, and verruga peruana (also called Peruvian warts), characterized by a widespread formation of hemangiomas (verrugas) on the skin, along with a persistent bacteremia (*3*,*7*). The primary vectors of *B. bacilliformis* are *Pintomyia verrucarum* and *Lutzomyia peruensis* sand flies, which can be found in the Inter-Andean valleys of Peru, at altitudes ranging from 500 to 3,200 meters (*7*). 

Carrion’s disease is a neglected disease because of its focal occurrence (Andean valleys of Peru and, to a lesser extent, in Colombia and Ecuador) and challenges in establishing diagnosis (lack of resources and difficult access to endemic areas). The occurrence of the disease in nonendemic areas and the detection of *B. bacilliformis* DNA in a growing range of sand fly species suggests that the epidemiologic cycle of Carrion’s disease might involve more sand fly species than first suspected (*19*). Researchers have detected *B. bacilliformis* DNA in wild-captured *Pintomyia robusta* sand flies in the border region between Ecuador and Peru (unpub. data, A.R. Carrazco-Montalvo, https://doi.org/10.13140/RG.2.2.17645.00481) and in *Pintomyia maranonensis* sand flies in northern Peru (*20*), but data have yet to confirm their role as vectors. Other possible vectors of Carrion’s disease were noted in Colombia, including *Lutzomyia gomezi*, *Psychodopygus panamensis*, *Pintomyia serrana*, and most notably *Pintomyia columbiana* sand flies, because of their presence in areas of disease outbreaks (*21*,*22*); however, those observations lacked molecular confirmation of the presence of *Bartonella* DNA in those sand fly specimens. Other suggested vectors for transmission of *B. bacilliformis* include *Lutzomyia pescei*, *L. noguchii*, and *L. ayacuchensis* sand flies (*19*,*22*,*23*).

Reports have identified *Bartonella ancashensis*, a species closely related to *B. bacilliformis*, from blood samples of patients undergoing treatment for verruga peruana in the rural region of Ancash, Peru (*24*,*25*). Although that species has not been isolated from blood samples of patients with Oroya fever and seems to be less pathogenic than *B. bacilliformis*, co-infections can occur, given that the geographic distribution of *B. ancashensis* overlaps with *B. bacilliformis* (*4*,*25*). Still, no reports have elucidated the involvement of sand flies in the transmission cycle of *B. ancashensis*.

Brazil has a rich diversity of 304 phlebotomine sand fly species (89 endemic), classified within 19 genera, distributed across all 5 federative regions of Brazil: 218 species in the north, 155 in the midwest, 132 in the southeast, 129 in the northeast, and 49 in the south (*26*). Despite the diverse phlebotomine sand fly fauna present in Brazil and the proximity to regions endemic for or reporting cases of Carrion’s disease, previous studies have not investigated the occurrence of *Bartonella* spp. in those dipterans. However, studies from other countries have detected the presence of *Bartonella* sp. DNA in sand fly species that inhabit Brazil. In Peru, individual female *Pintomyia nevesi* and *Lutzomyia sherlocki* sand flies and pooled female *Nyssomyia whitmani* and *Psychodopygus hirsutus* sand fly tested positive for *Bartonella* sp. DNA, phylogenetically associated with *B. bacilliformis* and *Candidatus* Bartonella rondoniensis (*27*). Researchers in Mexico detected *Bartonella gltA* genotypes, which have been associated with a putative new lineage of *Bartonella* in sand flies, in females *Lutzomyia cruciata* and *Psathyromyia shannoni* sand fly (*28*). In this study, we investigated the occurrence and molecular identity of *Bartonella* spp. in sand flies collected in 7 states across the north and northeast regions of Brazil.

## Material and Methods

### Sand Fly Specimens and Studied Areas

We analyzed sand fly specimens collected during November 2017–December 2021, captured by using Shannon traps or traps designed by the Centers for Disease Control and Prevention set up in ecologic reserves and parks throughout Brazil. Locations included preserved forest areas in the cities of Xapuri and Rio Branco (Acre); Murici Ecologic Station (Alagoas); Pau Brasil National Park (Bahia); Ubajara National Park (Ceará); Tapajós National Forest (Pará); Dois Irmãos State Park (Pernambuco); and Viruá National Park (Roraima). We extracted DNA from dissected sand flies by using Invitrogen TRIzol Reagent (Thermo Fisher Scientific, https://www.thermofisher.com); specimens were without heads and 3 last abdominal segments, which were used for morphologic identification according to previously described taxonomic keys (*29*). We evaluated DNA concentration and quality (260/280 ratio) with the use of a spectrophotometer (Nanodrop; Thermo Fisher Scientific). We assessed the presence of potential PCR inhibitors by using a conventional PCR based on cytochrome c oxidase subunit 1 (*cox1*), an endogenous gene among invertebrates. We investigated the occurrence of *Bartonella* sp. DNA in a total of 634 individual sand fly DNA samples, which we classified into 44 species belonging to 14 genera, obtained from 7 different states across north and northeast Brazil (Table).

**Table:** Species and number of sand flies, including regions they were collected, after PCR screening for amplification of the endogenous gene *cox*1 for investigation of *Bartonella spp.* in phlebotomine sand flies, Brazil*

Genera, no.	Species, no.	State of sampling
***Bichromomyia*, 4**	*flaviscutellata*, 4	Acre
***Brumptomyia*, 12**	sp., 12	Acre
***Evandromyia*, 60**	*begonae*, 1	Acre
	*infraspinosa*, 1	Acre
	*saulensis*, 14	Acre
	*termitophila*, 1	Acre
	*walkeri*, 43	Acre
***Lutzomyia*, 46**	*longipalpis*, 27	Ceará
	*sherlocki*, 19	Acre
***Micropygomyia*, 2**	*trinidanensis*, 1	Acre
	sp., 1	Pará
***Nyssomyia*, 132**	*antunesi*, 76	Acre
	*shawi*, 15	Acre
	*umbratilis*, 28	Pará, n = 14; Pernambuco, n = 14
	*whitmani*, 12	Acre
	sp., 1	Acre
***Pintomyia*, 13**	*nevesi*, 5	Acre
	*serrana*, 6	Acre
	sp., 2	Bahia
***Pressatia*, 28**	*choti*, 15	Bahia
	sp., 13	Acre, n = 5; Bahia, n = 8
***Psathyromia*, 3**	*elizabethdorvalae*, 2	Acre
	sp., 1	Acre
***Psychodopygus*, 163**	*amazonensis*, 3	Acre
	*ayrozai*, 40	Alagoas, n = 2; Bahia, n = 8; Roraima, n = 30
	*carreirai*, 25	Acre, n = 22; Roraima, n = 3
	*chagasi*, 26	Alagoas, n = 2; Pará, n = 6; Roraima, n = 18
	*complexus*, 3	Alagoas, n = 2; Pará, n = 1
	*davisi*, 30	Acre, n = 24; Pará, n = 6
	*guyanensis*, 1	Pará
	*hirsutus*, 2	Alagoas, n = 1; Bahia, n = 1
	*lainsoni*, 2	Acre
	*llanosmartinsi*, 11	Acre
	*paraensis*, 17	Pará, n = 5; Roraima, n = 12
	*squamiventris*, 1	Roraima
	sp., 2	Acre, n = 1; Roraima, n = 1
***Sciopemyia*, 2**	*sordelli*, 2	Acre
***Trichophoromyia*, 106**	*ubiquitalis*, 1	Pará
	*viannamartins*, 65	Alagoas
	sp., 40	Acre, n = 24; Pará, n = 16
***Trichopygomyia*, 61**	*dasypodogeton*, 2	Acre
	*longispina*, 55	Bahia
	sp., 4	Bahia, n = 2; Roraima, n = 2
***Viannamyia*, 2**	*furcata*, 2	Acre

*All sand fly samples were used for PCR amplification and phylogenetic characterization of *Bartonella* spp.

### Molecular Assays

We conducted molecular screening for *Bartonella* spp. by using a quantitative real-time PCR (qPCR) based on a 243-bp fragment of the 16S-23S ribosomal DNA internal transcribed spacer (ITS). We performed all reactions in a final volume of 10 µL containing 2× qPCRBIO Probe Master Mix Buffer (PCR Biosystems, https://pcrbio.com), 1.2 µM of each primer and probe, 1 µL of DNA sample, and ultrapurified, sterilized water qsp (Appendix Table 1). For the construction of the standard curve of each reaction, we performed serial dilutions at different concentrations (10^7^–10^1^ copies) of a gBlock gene fragment encoding a 243-bp fragment of the ITS genic region of *Bartonella henselae* (GenBank accession no. L35101) (Integrated DNA Technologies, https://www.idtdna.com). We also used the gBlocksas positive controls. 

We determined the number of gene copies by the formula (XG/μL DNA/[gene block length, bp × 660]) × 6.22 × 10^23^ × gene copies/μL. We calculated the amplification efficiency (E) according to the slope of the standard curve by using the formula E = 10^−1/slope^. We evalutated each DNA sample in duplicate and retested in triplicate those samples that presented differences in Cq values >0.5. We considered a Cq value cutoff of 42 for negative results. We carried out reactions in a C1000-CFX96 thermocycler (Bio-Rad Laboratories, https://www.bio-rad.com), using ultrapurified, sterilized water as a negative control.

We noted samples revealed to be positive in the screening qPCR and characterized them by using conventional PCRs based on 8 different molecular markers: *gltA* (380–400 bp), (767 bp), *ftsZ* (515 bp), *groEL* (752 bp), *nuoG* (346 bp), *pap31* (564 bp), *rpoB* (825 bp), *ribC* (585–588 bp), and 16S-23S ITS (453–717 bp) (Appendix Table 1). 

### Purification and Phylogenetic Analyzes

We purified the amplicons obtained in the PCRs by using Wizard SV Gel and PCR Clean-Up System (Promega Corporation, https://www.promega.com). We submitted purified amplicons for Sanger sequencing in both directions (forward and reverse) at the Centro de Estudos do Genoma Humano e Células Tronco (University of São Paulo, São Paulo, Brazil) by using the BigDye Terminator v3.1 Cycle Sequencing Kit (Thermo Fisher Scientific). We assembled a consensus sequence for each sample by using Geneious Prime 2023.2 (Geneious, https://www.geneious.com) and BioEdit 7.2 (*30*) software programs.

We conducted BLASTn analyzes (https://blast.ncbi.nlm.nih.gov) to produce an alignment for each genetic region, by using the obtained sequences, closely related sequences, and reference sequences previously deposited in GenBank. We created alignments by using the MAFFT version 7 software (https://mafft.cbrc.jp/alignment/server/index.html) and trimmed by using BioEdit 7.2 software (*30*). For phylogenetic inferences, we performed a maximum-likelihood analysis, with 10^3^ ultraFast bootstrap replicates for each alignment, by using IQTREE2 1.6.12 software (http://www.iqtree.org). We chose the best-fitting evolutionary model for each alignment by using MrModeltest2 2.4 (MrModeltest 2.4; https://github.com/nylander/MrModeltest2) through the PAUP4* Version 4c software (https://paup.phylosolutions.com). We rooted (via outgroups) and edited the resulting phylogenetic trees by using FigTree 1.4.4 (https://tree.bio.ed.ac.uk/software/figtree) and iTOL version 5 (https://itol.embl.de) software programs.

## Results

The DNA extraction of individual specimens of sand flies by using TRIzol was satisfactory, yielding DNA concentrations ranging from 1 to 15 ng/µL. We were able to obtain positive samples in the *cox1* conventional PCR for all 634 (100%) specimens.

**Figure 1:**
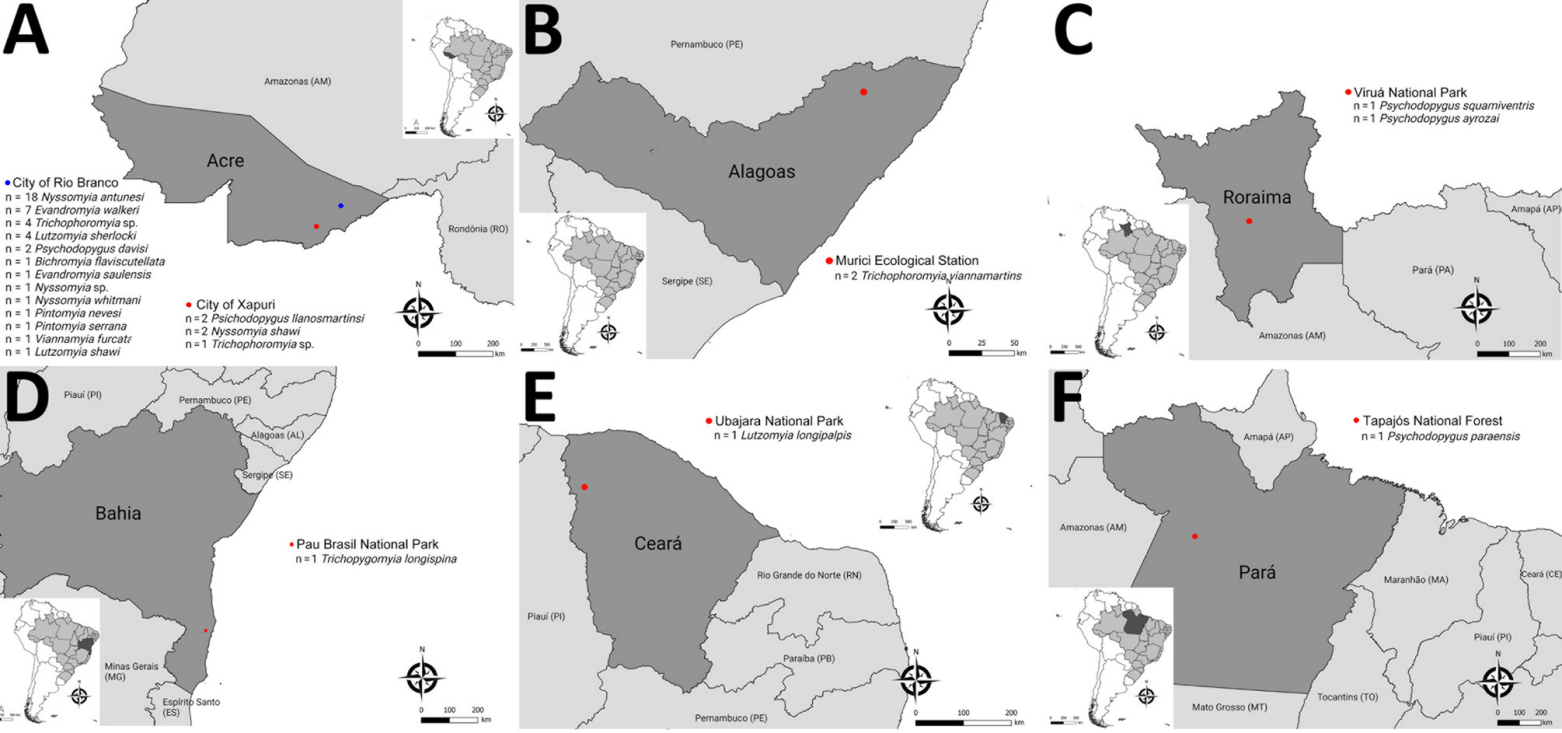
Sampling locations for sand flies that were qPCR positive in the screening for *Bartonella* spp. DNA from specimens collected in Brazil. A) State of Acre, northern Brazil; B) State of Alagoas, northeastern Brazil; C) State of Roraima, northern Brazil; D) State of Bahia, northeastern Brazil; E) State of Ceará, northeastern Brazil; F) State of Pará, northern Brazil. Dark gray indicates states with positive specimens, and red and blue dots representing the geographic location or city of sampling site. Inset maps show locations of each state in South America.

Using the qPCR assay targeting the 16S-23S ITS region, we found that 55 (8.67%) of 634 sand flies tested positive for *Bartonella* spp. in the molecular screening: 48 from Acre (*Nyssomyia antunesi* [n = 18], *Evandromyia walker*i [n = 7], *Trichophoromyia* sp. [n = 5], *L. sherlocki* [n = 4], *Nyssomyia shawi* [n = 3]; *Psychodopygus llanosmartinsi* [n = 2]; *Psychodopygus davisi* [n = 2]; *Bichromomyia flaviscutellata* [n = 1]; *Evandromyia saulensis* [n = 1]; *Nyssomyia* sp. [n = 1]; *N. whitmani* [n = 1]; *P. nevesi* [n = 1]; *Pintomyia serrana* [n = 1]; *Viannamyia furcata* [n = 1]); 2 from Alagoas (*Trichophoromyia viannamartinsi*); 2 from Roraima (*Psychodopygus squamiventris* [n = 1]; *Psychodopygus ayrozai* [n = 1]); 1 from Bahia (*Trichopygomyia longispina*); 1 from Ceará (*Lutzomyia longipalpis*); and 1 from Pará (*Psychodopygus paraensis*) (Figure 1). The range of Cq values of positive samples was 30.1–41.8. We selected 16 of those samples (based on the lowest PCR Cq values) and obtained 7 readable sequences. On the basis of BLASTn analysis, we confirmed that all 7 sequences corresponded to a *Bartonella* sp. (Appendix Table 2). However, the sequences were too short (179–222 bp) to be used for phylogenetic inferences. The value of the qPCR efficiency fell in the range of 98.7%–104.8% (mean 102.3, SD 2.29). The R^2^ value was 0.834–0.986 (mean 0.978, SD 0.05), the Y-intercept range was 34.429–42.318 (mean 37.97, SD, 2.75), and the slope was −3.22 to −3.35 (mean −3.27; SD, 0.05). We were unable to measure the DNA load of positive samples because the Cq difference between replicates was >0.5, possibly because of the Monte Carlo effect (*31*).

We performed further molecular characterization (by conventional PCR) of samples that tested positive in the ITS screening qPCR assay and generated amplicons for the following genes: 4 for the *gltA*, 4 for the ITS, 2 for the *ftsZ*, 2 for the *pap31*, 1 for the *rpoB*, and 1 for the *nuoG*. Of those, we obtained 2 readable sequences: one 377-bp *gltA* sequence (GenBank accession no. PP421218) from a *L. longipalpis* sand fly captured in the state of Ceará, and one 345-bp *nuoG* sequence from a *N. antunesi* sand fly from Acre.

**Figure 2:**
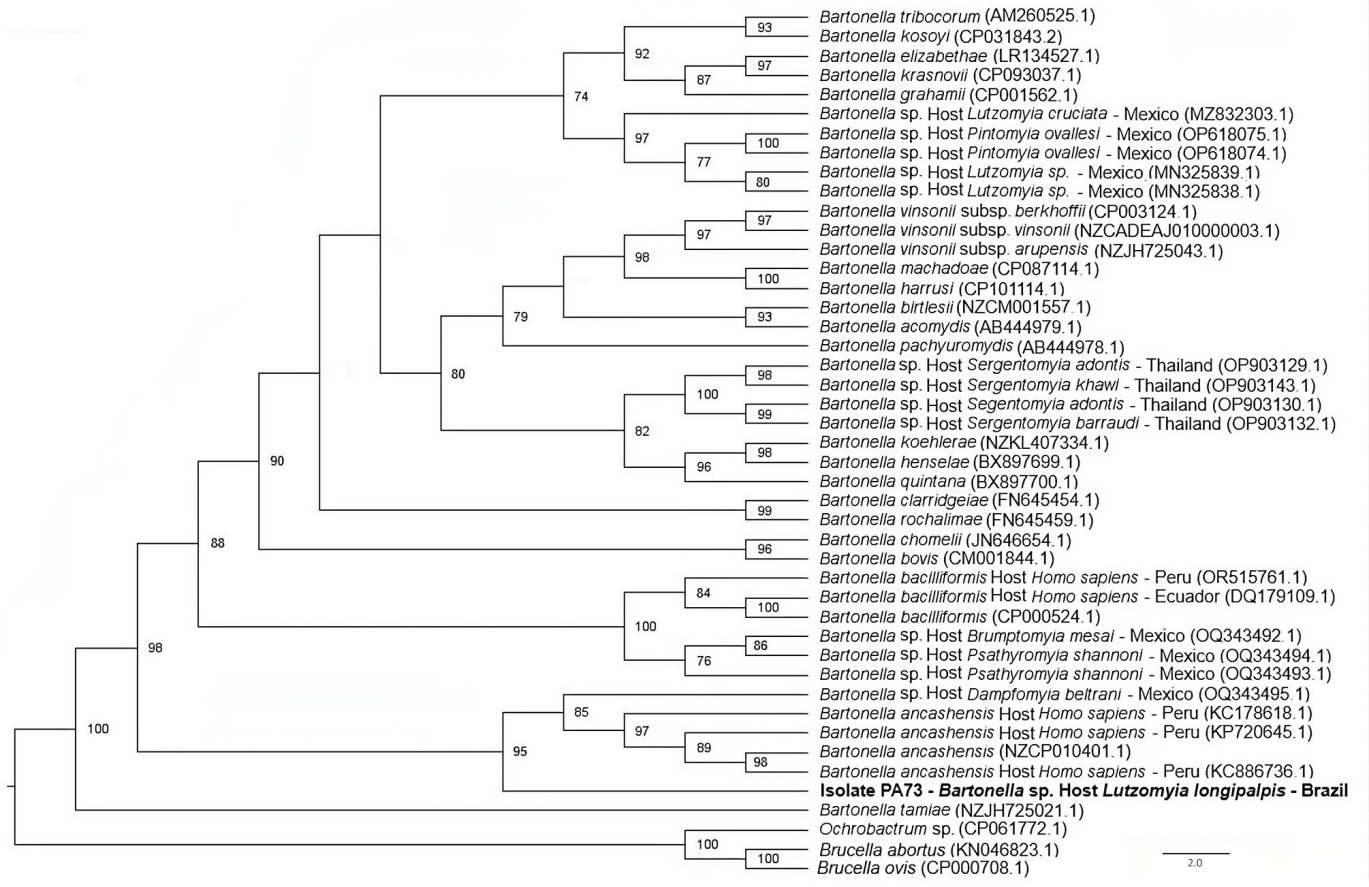
Phylogenetic tree based on an alignment of 380 bp-length of the *gltA* sequences obtained from phlebotomine sand flies collected in Brazil (bold) and reference sequences. Tree was created using the maximum-likelihood method and generalized time reversible plus invariate sites plus gamma as the evolutionary model. *Ochrobactrum* sp., *Brucella ovis*, and *Brucella abortus* were used as outgroups. Only bootstrap values >70 are shown. GenBank accession numbers are provided in parentheses.

The BLASTn analysis demonstrated that the *gltA* sequence obtained from *L. longipalpis* sand fly demonstrated >96% identity with *B. ancashensis* sequences previously obtained from infected humans (GenBank accession nos. CP010401.1, KC886736.1, and KC178618.1). Phylogenetic analyses positioned this sequence in the same subclade as *B. ancashensis* sequences and with a *Bartonella* sp. sequence detected in a *Dampfomyia beltrani* sand fly from Mexico (GenBank accession no. OQ343492.1), with a bootstrap clade support value of 95 (Figure 2).

The BLASTn analysis of the *nuoG* sequence from *N. antunesi* sand flies indicated a 94.04%–94.47% identity with 2 *Bartonella* sp. sequences obtained from *Pteronotus davyi* bats from Guatemala (GenBank accession nos. MN270091.1 and MN270098.1). The few *Bartonella nuoG* sequences in GenBank and low values of bootstrap clades hampered robust phylogenetic inference by using this molecular marker.

## Discussion

We documented the presence of *Bartonella* spp. DNA in phlebotomine sand flies from Brazil. The occurrence rate observed in this study (55/634 specimens; 8.67%) is similar that reported in southern Mexico, where 2 (8.69%) of 23 specimens were positive (*32*). Other investigations have reported a range of rates; 2 studies in Peru found positive results in 17 (6.02%) of 228 pools (*27*) and 2 (2.63%) of 76 pools (*20*), whereas 2 other studies in Mexico found positive results in 27 (5.08%) of 531 specimens (*33*) and 11 (2.06%) of 532 specimens (*28*). Differences in lower occurrence rates can be explained by the wide diversity of sand fly species present in different countries, the method of molecular analysis employed for DNA amplification, and, as illustrated in this study, technical limitations in obtaining phylogenetically relevant *Bartonella* DNA sequences from these small insects. Although the phlebotomine vectors of *Bartonella* spp. are very restricted to defined geographic areas, there have been minimal efforts to investigate the prevalence of this bacterial genus in sand flies from regions other than Peru. In our study, the selection of a broad diversity of sand fly species for *Bartonella* detection can be misleading, since most of the species are not confirmed to be carriers of these bacteria. In this context, we can assume that sand flies that were negative for the *Bartonella* sp. detection are either unable to host the bacteria or can be considered infrequent vectors. Further studies are necessary to elucidate the role of different sand fly species in the *Bartonella* epidemiologic cycles.

Although pooling specimens for analysis might have yielded a higher quantity of DNA (ng/µL), we would not have been able to accurately quantify the number of specimens that contained *Bartonella* sp. DNA, potentially leading to an underrepresentation of PCR-positive sand flies. Therefore, we opted to individually extract the DNA from the specimens by using the TRIzol reagent (Thermo Fisher Scientific), which resulted in satisfactory DNA quality, with concentrations of 1–15 ng/µL, and provided enough volume to perform the molecular detection and characterization. We confirmed the absence of PCR inhibitors by successfully amplifying the invertebrate *cox*1 gene in all samples.

Our detection of *Bartonella* sp. in *L. longipalpis* sand flies from Ceará state in northeastern Brazil corroborates previous findings. Our obtained 377-bp *Bartonella gltA* sequence clustered in the same subclade as *B. ancashensis* sequences obtained from humans with verruga peruana and a genotype recently detected in pools of *Dampfomyia beltrani* sand flies from Mexico (*34*). Of interest, genotypes closely related to *B. bacilliformis* were previously detected in *Psathyromyia* sand flies from Mexico, a nonendemic country for Carrion’s disease (*28*). Collectively, findings to date highlight the occurrence of putative novel genotypes belonging to ancient *Bartonella* lineages in sand flies from Brazil and Mexico, whose zoonotic potential remains unknown.

Although natural *Bartonella* sp. infections have not been previously reported in *L. longipalpis* sand flies, experimental studies of this species demonstrated infection with *B. ancashensis*, which remained viable in the anterior midgut for up to 7 days (*4*). A subsequent report describing the experimental infection of *L. longipalpis* sand flies with *B. bacilliformis* noted similar bacterial viability results (*35*). Although the *L. longipalpis* species has been used as a model for sand fly infection with *B. bacilliformis*, there are no reports of this species in Peru, where Carrion’s disease is endemic (*36*). Prior investigators have suggested *L. longipalpis* sand flies might play a short-term role in the maintenance of *Bartonella* and potentially serve as a vector during that time (*4*,*35*). Our data further reinforce the need for additional investigation into the potential role of various sand flies for transmission of *Bartonella* spp. to human patients and sick animals. Future research specifically focusing on *L. longipalpis* sand flies is of particular importance because the species is the main vector of *Leishmania infantum* and is widely distributed in Brazil and throughout Central and South America (*37*). Although absent from Peru, the *L. longipalpis* sand fly belongs to the same genus, albeit from different subgenus, as the primary vector of *Bartonella bacilliformis* in Peru, namely the *Lutzomyia (Helcocyrtomyia) peruensis* sand fly. Furthermore, the *L. longipalpis* sand fly is related to sand fly species in which *Bartonella* DNA have already been detected, namely *Lutzomyia (Tricholateralis) gomezi*, *Lutzomyia (Tricholateralis) cruciata*, *Lutzomyia (Tricholateralis) sherlocki*, or to species that have been incriminated as additional putative vectors for *B. bacilliformis*, namely *Lutzomyia (Helcocyrtomyia) pescei*, *Lutzomyia (Helcocyrtomyia) noguchii*, and *Lutzomyia (Helcocyrtomyia) ayacuchensis* (*7*,*19*,*22*,*23*,*29*; A.R. Carrazco-Montalvo, unpub. data). Those findings highlight the importance of the sand fly genus *Lutzomyia* sensu stricto in the transmission cycles of *Bartonella* in South America.

Our investigation also revealed the amplification of a *Bartonella* sp. *nuoG* sequence with ≈94% identity to sequences previously detected in insectivorous *P. davyi* bats from Guatemala. The obtained genotype (detected in a *Nyssomyia antunesi* specimen captured in the state of Acre) shared 88%–91% identity with other *Bartonella* sp. sequences previously detected in bats and their associated ectoparasites from Brazil, including sequences amplified from *Diphylla ecaudata* and *Desmodus rotundus* vampire bats (*38*) and *Trichobius dugesii* flies (*39*). Despite the diverse phlebotomine sand fly fauna found across many Brazil biomes (≈370 species) and the country’s proximity to regions reporting cases of Carrion’s disease and *Bartonella* sp. in sand flies, the occurrence of *Bartonella* in those dipterans in Brazil has been unconfirmed. However, based on phlebotomine sand fly feeding habits (*40*), many studies have reported the occurrence of *Bartonella* sp. in vertebrates that act as hosts for sand fly blood meals, including rodents (*41*,*42*), marsupials *43*), bats (*38*,*39*,*42*), and xenarthrans (*44*). Although Streblidae and Nycteribiidae flies act as the main putative vectors of *Bartonella* species transmission among bats (*39*,*45*), many sand fly species that feed on bats (and other hosts) can acquire *Bartonella* spp. infections during blood-feeding. We believe that sand fly feeding habits and the high prevalence of *Bartonella* infection in many reservoir mammal hosts indicates a potential relationship and involvement of sand flies in the epidemiologic cycles of these bacteria.

In conclusion, we amplified *Bartonella* spp. DNA and successfully sequenced from *L. longipalpis* and *Ny. antunesi* sand flies, indicating possible involvement of these phlebotomine species in the maintenance or transmission cycle of *Bartonella* spp. The *Bartonella gltA* genotype was closely related to *B. ancashensis*, and the *nuoG* genotype was most closely related to a bat-associated *Bartonella* sp. Determining the epidemiologic cycle of these agents in Brazil will require elucidating the species and lineages of *Bartonella* spp. circulating among sand flies and determining whether sand flies in Brazil are capable of *Bartonella* spp. transmission to animals, including humans.

Appendix More information for *Bartonella* spp. in phlebotomine sand flies, Brazil.
