# Supplementary material for: Bartonella spp. in Phlebotominae Sand Flies, Brazil
Source: Emerg Infect Dis. 2024 Oct;30(10):2099–107. doi: 10.3201/eid3010.240397 (PMC11431920; doi:10.3201/eid3010.240397)
Supplement: Appendix — More information for Bartonella spp. in phlebotomine sand flies, Brazil. [file 24-0397-Techapp-s1.pdf]

Article DOI: https://doi.org/10.3201/eid3010.240397

EID cannot ensure accessibility for supplementary materials supplied by authors. Readers who have difficulty accessing supplementary content should contact the authors for assistance.

Bartonella spp. in Phlebotomine Sand Flies, Brazil

Appendix

Appendix Table 1. Description of molecular assays (target gene, primers/probe identification and sequence, thermal conditions, amplicon size and reference) used for sand flies' endogenous gene screening and characterization of *Bartonella* spp. in the present study.

Molecular Marker	Primers/Probe	Thermal conditions	Amplicon size (bp)	Reference
cox-1	**LCO1490**<br>(5′ -GGTCAACAAATCATAAAGATATTGG- 3′)<br>**HCO2198**<br>(5′ -TAAACTTCAGGGTGACCAAAAAATCA- 3′)	95°C for 1min; 35 cycles of 95°C for 1min, 40°C for 1min and 72°C for 1.5min; 72°C for 10min	710	(*1*)
16S-23S ITS	**BsppITS325S Forward**<br>(5′ – CTTCAGATGATGATCCCAAGCCTTCTGGCG – 3′)<br>**543as Reverse**<br>(5′ – AATTGGTGGGCCTGGGAGGACTTG – 3′)<br>**BsppITS500**<br>(5′ – [6FAM]GTTAGAGCGCGCGCTTGATAAG[BHQ1] – 3′)	95°C for 3min; 45 cycles of 94°C for 10s, 68°C for 10s, 72°C for 10s and plate read; 72°C for 30s	243	(*2*,*3*)
gltA	**BhCS.781p (gltA F)**<br>(5′ GGGGACCAGCTCATGGTGG- 3′)<br>**BhCS.1137n (gltA R)**<br>(5′ AATGCAAAAAGAACAGTAAACA- 3′)	95°C for 5min; 35 cycles of 95°C for 30s, 51°C for 30s and 72°C for 2min; 72°C for 5min	380–400	(*4*)
	**CS443f**<br>(5′ -GCTATGTCTGCATTCTATCA- 3′)<br>**CS1210r**<br>(5′ -GATCYTCAATCATTTCTTTCCA- 3′)	94°C for 2min; 45 cycles of 94°C for 30s, 48°C for 1min and 72°C for 1min; 72°C for 7min	767	(*5*)
ftsZ	**ftsZ F**<br>(5′ -CATATGGTTTTCATTACTGCYGGTATGG- 3′)<br>**ftsZ R**<br>(5′ -TTCTTCGCGAATACGATTAGCAGCTTC- 3′)	94°C for 2min; 40 cycles of 94°C for 2min, 61°C for 45s and 72°C for 45s; 72°C for 7min	515	(*6*)
groEL	**GroEL F**<br>(5′ -GGAAAAAGTGGGCAATGAAG- 3′)<br>**GroEL R**<br>(5′ -TCCTTTAACGGTCAACGCATT- 3′)	94°C for 2min; 40 cycles of 94°C for 2min, 47°C for 45s and 72°C for 45s; 72°C for 7min	752	(*6*)
nuoG	**nuoG F**<br>(5′ -GGCGTGATTGTTCTCGTTA- 3′)	94°C for 5min; 35 cycles of 94°C for 30s, 53°C for 30s and 72°C for 5min; 72°C for 5min	346	(*7*)

Molecular Marker	Primers/Probe	Thermal conditions	Amplicon size (bp)	Reference
	**nuoG R**<br>(5′ -CACGACCACGGCTATCAAT −3′)			
Pap31	**165s**<br>(5′ -GACTTCTGTTATCGCTTTGATTT- 3′)<br>**688as**<br>(5′ -CACCACCAGCAAMATAAGGCAT- 3′)	95°C for 5min; 45 cycles of 94°C for 30s, 56°C for 30s and 72°C for 45s; 72°C for 5min	564	(*8*)
rpoB	**1400F**<br>(5′ -CGCATTGGCTTACTTCGTATG- 3′)<br>**2300R**<br>(5′ -GTAGACTGATTAGAACGCTG- 3′)	94°C for 2min; 35 cycles of 94°C for 30s, 53°C for 30s and 72°C for 1min; 72°C for 2min	825	(*9*)
ribC	**Barton-1**<br>(5′ -TAACCGATATTGGTTGTGTTGAAG- 3′)<br>**Barton-2**<br>(5′ -TAAAGCTAGAAAGTCTGGCAACATAACG- 3′)	95°C for 10min; 37 cycles of 95°C for 1min, 51°C for 1min and 72°C for 1min; 72°C for 3min	585–588	(*10*)
16S-23S ITS	**321s**<br>(5′ -AGATGATGATCCCAAGCCTTCTGGCG- 3′)<br>**938as**<br>(5′ -TGTTCTYACAACAATGATGATG- 3′)	95°C for 5min; 45 cycles of 94°C for 30s, 54°C for 30s and 72°C for 45s; 72°C for 5min	453–717	(*11*)

Appendix Table 2. Sequence identity of the 16S-23S ITS positive samples to the most closely related *Bartonella* species.

ID	Sand fly species	Sequence size (bp)	BLASTn result	Host	Query Cover	E value	Identity	Country	Accession Number
AB121	*Trichophoromyia* sp.	222	*Bartonella ancashensis*	*Homo sapiens*	98%	9e-63	87.56%	Peru	KC886740.1
AB410	*Nyssomyia antunesi*	197	*Bartonella* sp.	*Mimon cozumelae*	81%	4e-71	98.16%	Guatemala	MN504740.1
			Bartonella sp.	*Micronycteris microtis*	81%	4e-71	98.16%	Guatemala	MN258132.1
AB594	*Psychodopygus davisi*	189	*Bartonella* sp.	*Mimon cozumelae*	87%	2e-78	100%	Guatemala	MN504740.1
			Bartonella sp.	*Micronycteris microtis*	87%	2e-78	100%	Guatemala	MN258132.1
AB164	*Psychodopygus llanosmartinsi*	189	*Bartonella* sp.	*Apodemus sylvaticus*	87%	1e-60	93.49%	United Kingdom	AJ269793.1
AB537	*Evandromyia walker*	179	*Bartonella* sp.	*Mimon cozumelae*	89%	4e-75	99.38%	Guatemala	MN504740.1
			Bartonella sp.	*Micronycteris microtis*	89%	4e-75	99.38%	Guatemala	MN258132.1
AB540	*Pintomyia serrana*	175	*Bartonella* sp.	*Apodemus sylvaticus*	100%	1e-60	92.09%	United Kingdom	AJ269793.1
AB12	*Nyssomyia antunesi*	197	*Bartonella* sp.	*Apodemus sylvaticus*	89%	1e-65	93.3%	United Kingdom	AJ269793.1

References

1. Folmer O, Black M, Hoeh W, Lutz R, Vrijenhoek R. DNA primers for amplification of mitochondrial cytochrome c oxidase subunit I from diverse metazoan invertebrates. Mol Mar Biol Biotechnol. 1994;3:294–9. PubMed

2. Breitschwerdt EB, Maggi RG. *Bartonella quintana* and *Bartonella vinsonii* subsp. *vinsonii* bloodstream co-infection in a girl from North Carolina, USA. Med Microbiol Immunol. 2019;208:101–7. PubMed https://doi.org/10.1007/s00430-018-0563-0

3. Oteo JA, Maggi R, Portillo A, Bradley J, García-Álvarez L, San-Martín M, et al. Prevalence of *Bartonella* spp. by culture, PCR and serology, in veterinary personnel from Spain. Parasit Vectors. 2017;10:553. PubMed https://doi.org/10.1186/s13071-017-2483-z

4. Norman AF, Regnery R, Jameson P, Greene C, Krause DC. Differentiation of *Bartonella*-like isolates at the species level by PCR-restriction fragment length polymorphism in the citrate synthase gene. J Clin Microbiol. 1995;33:1797–803. PubMed https://doi.org/10.1128/jcm.33.7.1797-1803.1995

5. Billeter SA, Gundi VAKB, Rood MP, Kosoy MY. Molecular detection and identification of Bartonella species in *Xenopsylla cheopis* fleas (Siphonaptera: Pulicidae) collected from *Rattus norvegicus* rats in Los Angeles, California. Appl Environ Microbiol. 2011;77:7850–2. PubMed https://doi.org/10.1128/AEM.06012-11

6. Paziewska A, Harris PD, Zwolińska L, Bajer A, Siński E. Recombination within and between species of the alpha proteobacterium *Bartonella* infecting rodents. Microb Ecol. 2011;61:134–45. PubMed https://doi.org/10.1007/s00248-010-9735-1

7. Colborn JM, Kosoy MY, Motin VL, Telepnev MV, Valbuena G, Myint KS, et al. Improved detection of *Bartonella* DNA in mammalian hosts and arthropod vectors by real-time PCR using the NADH dehydrogenase gamma subunit (*nuoG*). J Clin Microbiol. 2010;48:4630–3. PubMed https://doi.org/10.1128/JCM.00470-10

8. Maggi RG, Breitschwerdt EB. Isolation of bacteriophages from *Bartonella vinsonii* subsp. *berkhoffii* and the characterization of *Pap31* gene sequences from bacterial and phage DNA. J Mol Microbiol Biotechnol. 2005;9:44–51. PubMed

9. Renesto P, Gouvernet J, Drancourt M, Roux V, Raoult D. Use of rpoB gene analysis for detection and identification of *Bartonella* species. J Clin Microbiol. 2001;39:430–7. PubMed https://doi.org/10.1128/JCM.39.2.430-437.2001

10. Johnson G, Ayers M, McClure SCC, Richardson SE, Tellier R. Detection and identification of *Bartonella* species pathogenic for humans by PCR amplification targeting the riboflavin synthase gene (*ribC*). J Clin Microbiol. 2003;41:1069–72. PubMed https://doi.org/10.1128/JCM.41.3.1069-1072.2003

11. Maggi RG, Breitschwerdt EB. Potential limitations of the 16S-23S rRNA intergenic region for molecular detection of *Bartonella* species. J Clin Microbiol. 2005;43:1171–6. PubMed https://doi.org/10.1128/JCM.43.3.1171-1176.2005
